# Smoking Relapse and Type 2 Diabetes Mellitus–Related Emergency Department Visits Among Senior Patients with Diabetes

**DOI:** 10.5888/pcd16.190027

**Published:** 2019-12-19

**Authors:** Yu-Hsiang Kao, Michael D. Celestin, Carl D. Walker, Qingzhao Yu, John Couk, Sarah Moody-Thomas, Huijie Zhang, Tung-Sung Tseng

**Affiliations:** 1 Behavioral and Community Health Sciences, School of Public Health, Louisiana State University Health Sciences Center New Orleans, New Orleans, Louisiana; 2 Health Care Services Division, Louisiana State University Health Sciences Center New Orleans, New Orleans, Louisiana; 3 Biostatistics, School of Public Health, Louisiana State University Health Sciences Center New Orleans, New Orleans, Louisiana; 4 Nafang Hospital of Southern Medical University, Guangzhou, Guangzhou, China

## Abstract

**Introduction:**

Quitting smoking has been proven to benefit smokers with diabetes. However, among older patients with diabetes, the evidence regarding an association between smoking status and the risk of type 2 diabetes mellitus–related emergency department (ED) visits has not been well investigated.

**Methods:**

A retrospective cohort study was performed by using the Louisiana State University Health Care Services Division electronic health records from 2009 to 2011. Patients aged 65 years or older with type 2 diabetes and smoking status recorded at least twice in 2010 were selected. Selected patients with diabetes were classified into nonsmokers, former smokers, continuing smokers, and relapsed smokers. Cox proportional hazards regression models were used to estimate the adjusted hazard ratio (aHR) of 1-year type 2 diabetes–related ED visits for each group compared with nonsmokers.

**Results:**

There were 174 (8.2%) continuing smokers and 77 (3.6%) relapsed smokers in 2,114 patients with diabetes who were studied. Rates of type 2 diabetes–related ED visits were highest in relapsed smokers (28.6%). Compared with nonsmokers, relapsed smokers had a significantly higher risk of type 2 diabetes–related ED visits (aHR = 1.62; 95% confidence interval [CI], 1.04–2.50). After stratifying by sex, a significantly increased risk of type 2 diabetes–related ED visits was shown only in male relapsed smokers (aHR = 2.05; 95% CI, 1.13–3.71) and female continuing smokers (aHR = 1.65; 95% CI, 1.10–2.47) compared with nonsmokers.

**Conclusion:**

Older men with diabetes who were relapsed smokers had a higher risk of type 2 diabetes–related ED visits. Future research and clinical practice should focus on these patients and create more effective interventions for smoking cessation and diabetes management.

**Summary**

**What is already known on this topic?**

People with diabetes who smoke are more likely to have serious health problems compared with their nonsmoking counterparts. The American Diabetes Association guidelines suggest that patients with diabetes quit smoking to improve diabetes management.

**What is added by this report?**

We identified the patient’s smoking status in the past 30 days and in the past year by using electronic health records. Among older patients with diabetes, continuing and relapsed smokers with diabetes had a higher risk for type 2 diabetes mellitus–related emergency department (ED) visits.

**What are the implications for public health practice?**

The results of our study suggest that older smokers with diabetes should remain abstinent from smoking to decrease type 2 diabetes–related ED visits.

## Introduction

Type 2 diabetes mellitus, a highly prevalent disease, is a major cause of illness and death worldwide (1). People with diabetes are more likely to use health care services, including inpatient, outpatient, and emergency department (ED), than those without diabetes (2). According to the 2017 National Diabetes Statistics Report, 30.3 million Americans had diabetes, of whom 12 million were aged 65 years or older (3). Older patients with diabetes have higher mortality (4) and ED use than younger patients with diabetes (5) and have approximately double the health care expenditures compared with their younger counterparts (6). Moreover, older patients with diabetes have a much higher rate of diabetes-related ED visits than younger patients with diabetes (aged 18–44 or 45–64 years) (5). Hence, research on older patients with diabetes is needed now because of the aging society.

Smoking is a risk factor for developing diabetes (7). People with diabetes who smoke are more likely to have serious health problems compared with their nonsmoking counterparts. Smokers with diabetes have a higher risk of developing serious complications (7), worse metabolic control (8), and periodontal inflammatory conditions (9), which likely lead to a lower quality of life and increased mortality (10). Benefits of smoking cessation have been demonstrated in smokers with diabetes, such as better glycemic control if they quit smoking (11). Therefore, the American Diabetes Association guidelines strongly suggest that patients with diabetes quit smoking to improve diabetes management, even if they are older than 65 (12). However, the association between smoking and diabetes-related ED visits for older patients with diabetes is not well understood. Therefore, the primary objective for this study was to investigate the association between smoking and the likelihood of diabetes-related ED visits among older patients with diabetes.

## Methods

This study applied a retrospective study design by using the Louisiana State University (LSU) Health Care Services Division (HCSD) data sets from 2009 to 2011. The LSU HCSD, which operates 7 public hospitals and clinics in Louisiana, is the largest provider of health care to Louisiana’s uninsured and low-income citizens (13). Approximately 60% of adult patients in the LSU HCSD outpatient clinic population are uninsured; in addition, 45% of all adult clinic patients are eligible for free care under Louisiana law. Free care eligibility is determined by household income and household size, with eligibility available to patients from households falling below 200% of the federal poverty guidelines (13). The electronic health record (EHR) (14) is a tool that has demonstrated great efficiency as part of an integrated approach to not only support caregivers’ decisions but also improve patients’ outcomes and can be used to assist with tobacco use intervention (13,15). The benefits of the EHR for providing recommendations for clinical action steps on tobacco use cessation have been established (16). For example, documentation of tobacco use status and referral to cessation counseling have been shown to increase after using the EHR to record and treat patient tobacco use at medical visits (17). Our study protocol was reviewed and approved by the Institutional Review Board of LSU HCSD—New Orleans.

### Study subjects

**Figure 1:**
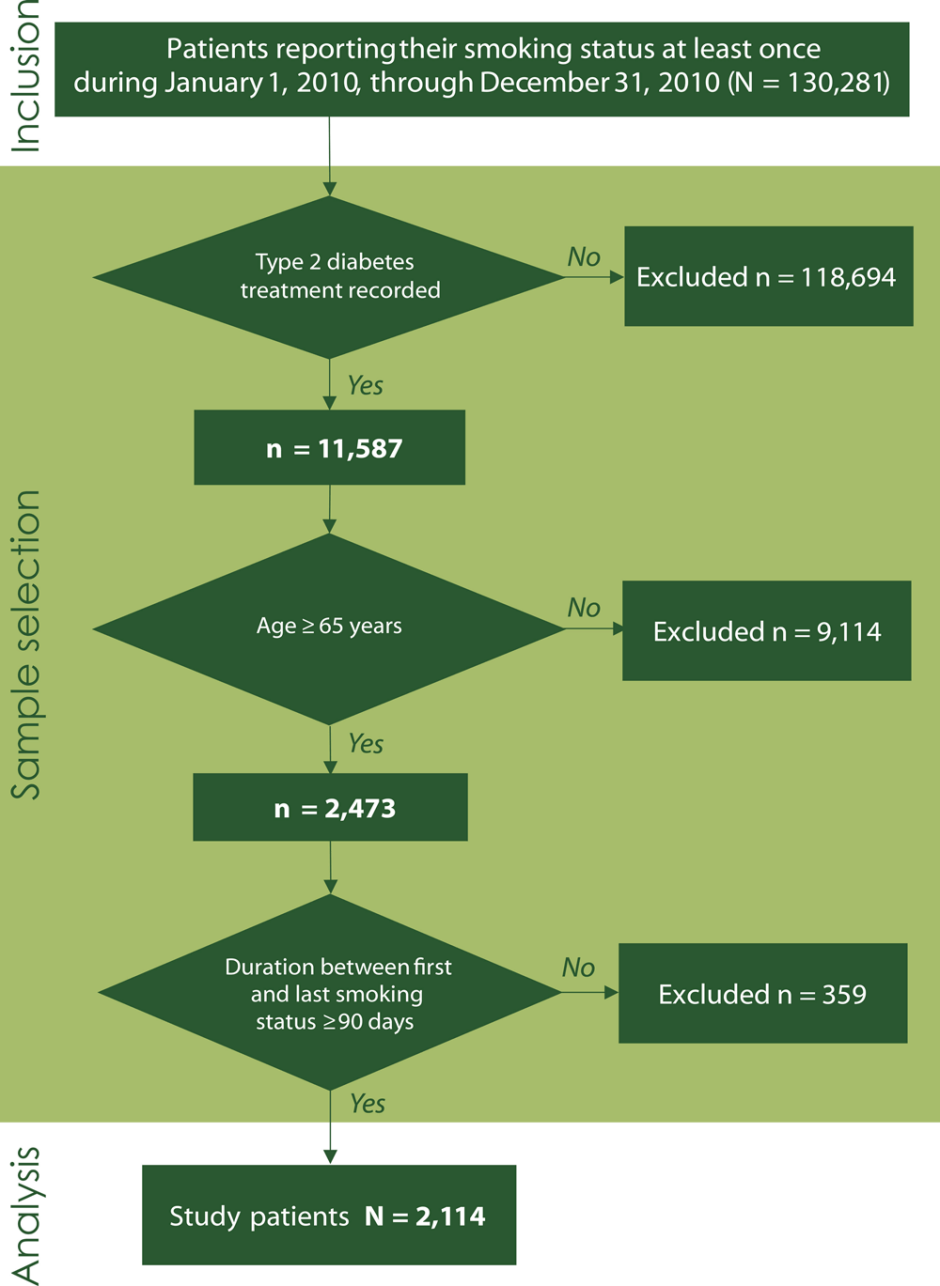
Sample selection for study of type 2 diabetes mellitus and smoking, Louisiana State University Health Care Services Division, 2009–2011.

A total of 130,281 patients who had at least 1 smoking status record (has or has not smoked in past 30 days) in 2010 in the LSU HCSD database were selected. The index date was set as the latest date for patients who had a record that demonstrated smoking status. Patients who did not have at least 2 ambulatory visits or 1 inpatient admission for type 2 diabetes mellitus (18) as a major diagnosis (*International Classification of Diseases, Ninth Revision, Clinical Modification* [ICD-9-CM], codes 250.x0 and 250.x2) (19) in the preceding year of the index date were excluded (n = 118,694). According to Centers for Medicare and Medicaid Services (CMS) Chronic Conditions Data Warehouse condition algorithms, qualified patients with diabetes had had at least 2 outpatient visits or at least 1 inpatient visit with a diabetes diagnosis (18). Additionally, when considering patients with 2 outpatient visits with diabetes, a prior study stated it may be helpful to exclude people who might be coded while they are suspected of having diabetes but were not formally diagnosed or were miscoded (19). Furthermore, patients aged less than 65 years at the index date (n = 9,114) were excluded. Lastly, 359 patients were excluded because the duration between the first and last record of smoking status was less than 90 days. In total, 2,114 patients were included (Figure 1).

### Assessment of smoking status

**Figure 2:**
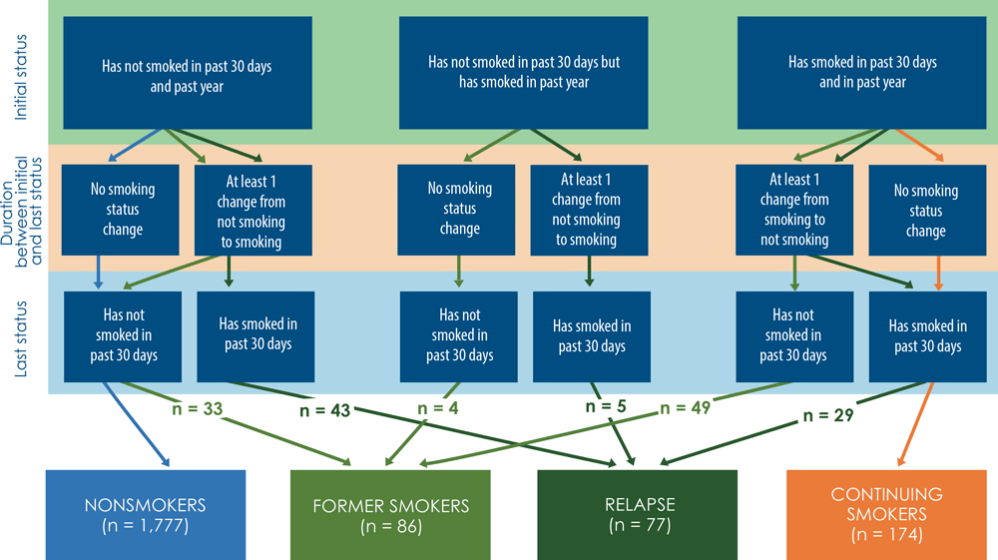
Patterns of identifying smoking status among patients with type 2 diabetes mellitus, Louisiana State University Health Care Services Division, 2009–2011. There was no significant difference in days of duration between initial and last status across 4 smoking status groups (*P* = .667).

Based on the patient’s smoking status in the past 30 days and in the past year, smoking status was classified into 4 types: nonsmoker, former smoker, continuing smoker, and relapsed smoker (Figure 2). Nonsmokers were patients who did not have a record of smoking at the initial time point (past 30 days and past year), last time point (past 30 days), and in the duration between the 2 time points (n = 1,777). Continuing smokers were patients who reported that they had smoked at the initial time point (past 30 days and past year), last time point (past 30 days), and all records in the duration between the initial and last time point (n = 174). Former smokers (n = 86) were patients with 1 of the following 3 conditions: 1) reported that they had smoked at the initial time point (past 30 days or past year), had not smoked at the last time point (past 30 days), and had at least 1 change from smoking to not smoking in the duration between the initial and last time point (n = 49); 2) reported that they had not smoked at the initial time point (past 30 days or past 1 year), had not smoked at the last time point (past 30 days), and had at least 1 change from not smoking to smoking in the duration between the initial and last time point (n = 33); or 3) reported that they had not smoked at the initial time point (past 30 days) but smoked in the past year, had not smoked at the last time point (past 30 days), and had no changes in the duration between the initial and last time point (n = 4). Relapsed smokers (n = 77) were patients with 1 of the following 3 conditions: 1) reported that they had not smoked at the initial time point (past 30 days or past 1 year), had smoked at the last time point (past 30 days), and had at least 1 change from not smoking to smoking in the duration between the initial and last time point (n = 43); 2) reported that they had not smoked at the initial time point (past 30 days or past year), had smoked at the last time point (past 30 days), and had at least 1 change from smoking to not smoking in the duration between the initial and last time point (n = 29); or 3) reported that they had not smoked at the initial time point (past 30 days) but smoked in the past year, had smoked at the last time point (past 30 days), and had at least 1 change from not smoking to smoking in the duration between the initial and last time point (n = 5).

### Emergency department use for diabetes

The outcome in our study concerned diabetes-related ED visits which were identified by principal ICD-9-CM diagnostic codes (250.00, 250.10, 250.20, 250.30, 250.40, 250.50, 250.60, 250.70, 250.80, 250.90, 250.02, 250.12, 250.22, 250.32, 250.42, 250.52, 250.62, 250.72, 250.82, and 250.92) (19). An ED visit was defined as an event that occurred during the outcome period. The follow-up duration was defined as the number of days from the study index date to the date of the end of a diabetes-related ED visit. If a patient had no ED visit for diabetes, then that patient was removed at the end of the 1-year follow-up period.

### Covariables

Numerous control variables were considered in this study. Patient characteristics used in this study were age, sex, race (20), and insurance type (commercial, free care, Medicare, Medicaid, and self-pay) (21). Although patient educational level or household income may have affected health care outcomes (20), the HCSD claims data set did not provide this information. Therefore, insurance type was used as a proxy for socioeconomic status. In addition, health status and comorbidity may affect ED use (20); therefore, the Charlson comorbidity index (CCI) (21), number of ambulatory visits and hospitalizations for diabetes (21), congestive heart failure (CHF), peripheral vascular disease (PVD), cerebrovascular disease (CVD), and chronic pulmonary disease (CPD) (5,22) were included if they occurred during the year before the index date. CCI was calculated for each patient according to outpatient or inpatient care by using the Quan adaptation of the Elixhauser comorbidities (22).

### Statistical analysis

The association of patient characteristics with smoking status was examined by using a χ^2^ test for categorical variables and a 1-way analysis of variance for continuous variables. Cox proportional hazards regression models were used to examine the association between smoking status and the risk of diabetes-related ED visit in older patients with diabetes. In the adjusted model, we calculated the adjusted hazard ratio (aHR) after adjusting for sex, age, race, insurance type, number of ambulatory visits for diabetes, hospitalization for diabetes, CCI, CHF, PVD, CVD, and CPD. No variable had multicollinearity in the adjusted model. Furthermore, we stratified by sex to investigate the association between smoking status and diabetes-related ED visits that may be attributable to sex differences in smoking cessation (23). In addition, the proportional hazard assumption was tested for all models (24). All analyses were performed by using SAS version 9.4 (SAS Institute Inc). All tests were 2-sided, and a *P* value of less than .05 was defined as significant.

## Results

Across the entire sample (N = 2,114), the mean age was 71.0 years (standard deviation [SD], 5.6), and 64.2% of the patients were women (Table 1). There were 1,777 (84.1%) patients identified as nonsmokers, 86 (4.1%) as former smokers, 174 (8.2%) as continuing smokers, and 77 (3.6%) as relapsed smokers. Almost half (48.5%) of the patients were white, and 73.9% of the patients had Medicare. Regarding medical conditions, 32.8% of the patients had had 5 or more outpatient visits for diabetes; patients had histories of CHF (8.5%), PVD (2.5%), CVD (1.8%), and CPD (6.7%). The overall incidence of diabetes-related ED visits was 19.6%, and the rate among patients who were relapsed smokers and continuing smokers were 28.6% and 22.4%, respectively (Table 1).

**Table 1:** Characteristics of Study Population (N = 2,114) by Smoking Status, Louisiana State University Health Care Services Division, 2009–2011^a^

Variables	Overall	Nonsmoker	Former Smoker	Continuing Smoker	Relapsed Smoker	*P* Value^b^
**Overall**	2,114 (100.0)	1,777 (84.1)	86 (4.1)	174 (8.2)	77 (3.6)	<.001
**Age, mean (SD), y**	71.0 (5.6)	71.4 (5.8)	70.4 (5.4)	68.8 (4.0)	68.7 (3.5)	<.001^c^
**Sex**						
Female	1,358 (64.2)	1,168 (65.7)	48 (55.8)	99 (56.9)	43 (55.8)	.01
Male	756 (35.8)	609 (34.3)	38 (44.2)	75 (43.1)	34 (44.2)	
**Race**						
African American	1,022 (48.3)	856 (48.2)	51 (59.3)	75 (43.1)	40 (52.0)	.18
White	1,026 (48.5)	864 (48.6)	33 (38.4)	92 (52.9)	37 (48.1)	
Other	66 (3.1)	57 (3.2)	2 (2.3)	7 (4.0)	0	
**Insurance type**						
Commercial	336 (15.9)	278 (15.6)	14 (16.3)	34 (19.5)	10 (13.0)	.14^d^
Free care	163 (7.7)	135 (7.6)	5 (5.8)	16 (9.2)	7 (9.1)	
Medicaid	28 (1.3)	21 (1.2)	1 (1.2)	6 (3.5)	0	
Medicare	1,563 (73.9)	1,323 (74.5)	63 (73.3)	117 (67.2)	60 (77.9)	
Self-pay	24 (1.1)	20 (1.1)	3 (3.5)	1 (0.6)	0	
**Type 2 diabetes mellitus outpatient visits**						
0–4	1,420 (67.2)	1,202 (67.6)	51 (59.3)	115 (66.1)	52 (67.5)	.44
≥5	694 (32.8)	575 (32.4)	35 (40.7)	59 (33.9)	25 (32.5)	
**Type 2 diabetes mellitus inpatient visits**						
0	2,104 (99.5)	1,768 (99.5)	86 (100.0)	173 (99.4)	77 (100.0)	.82^d^
≥1	10 (0.5)	9 (0.5)	0 (0.0)	1 (0.6)	0	
**Charlson Comorbidity Index^e^**						
0	1,402 (66.3)	1,184 (66.6)	56 (65.1)	116 (66.7)	46 (59.7)	.11
1	255 (12.1)	202 (11.4)	11 (12.8)	31 (17.8)	11 (14.3)	
≥2	457 (21.6)	391 (22.0)	19 (22.1)	27 (15.5)	20 (26.0)	
**Congestive heart failure**						
No	1,934 (91.5)	1,619 (91.1)	81 (94.2)	163 (93.7)	71 (92.2)	.52
Yes	180 (8.5)	158 (8.9)	5 (5.8)	11 (6.3)	6 (7.8)	
**Peripheral vascular disease**						
No	2,061 (97.5)	1,736 (97.7)	85 (98.8)	166 (95.4)	74 (96.1)	.18^d^
Yes	53 (2.5)	41 (2.3)	1 (1.2)	8 (4.6)	3 (3.9)	
**Cerebrovascular disease**						
No	2,075 (98.2)	1,751 (98.5)	83 (96.5)	168 (96.6)	73 (94.8)	.012^d^
Yes	39 (1.8)	26 (1.5)	3 (3.5)	6 (3.5)	4 (5.2)	
**Chronic pulmonary disease**						
No	1,972 (93.3)	1,670 (94.0)	78 (90.7)	154 (88.5)	70 (90.9)	.02
Yes	142 (6.7)	107 (6.0)	8 (9.3)	20 (11.5)	7 (9.1)	
**Emergency department visit for type 2 diabetes mellitus**						
No	1,700 (80.4)	1,439 (81.0)	71 (82.6)	135 (77.6)	55 (71.4)	.14
Yes	414 (19.6)	338 (19.0)	15 (17.4)	39 (22.4)	22 (28.6)	

a All data are number (percentage) unless otherwise indicated.

b χ^2^ test unless otherwise indicated.

c Analysis of variance.

d Fisher exact test.

e Reference 21.

After adjustment, relapsed smokers had a significantly higher risk of diabetes-related ED visits (aHR, 1.62; 95% confidence interval [CI], 1.04–2.50) (Table 2). Despite there being no significant difference for continuing smokers compared with nonsmokers, results showed an increased risk of diabetes-related ED visits for continuing smokers, for both crude (cHR, 1.19; 95% CI, 0.85–1.65) and adjusted (aHR, 1.19; 95% CI, 0.85–1.67) models. Compared with patients with fewer than 5 ambulatory visits for diabetes, patients with 5 or more outpatient visits in the preceding year were 36% more likely to have an ED visit for diabetes (aHR, 1.36; 95% CI, 1.11–1.66). In addition, the risk of diabetes-related ED visits was associated with a significant increase among patients who had a comorbidity such as CHF (aHR, 1.77; 95% CI, 1.25–2.52) and CPD (aHR, 1.92; 95% CI, 1.32–2.80).

**Table 2:** Factors Associated With Emergency Department Use for Type 2 Diabetes Mellitus Using Cox Models (N = 2,114), Louisiana State University Health Care Services Division, 2009–2011

Factor	Crude Hazard Ratio (95% Confidence Interval)	Adjusted Hazard Ratio (95% Confidence Interval)
**Smoking status (reference: nonsmoker)**		
Former smoker	0.94 (0.56–1.58)	0.91 (0.54–1.52)
Continuing smoker	1.19 (0.85–1.65)	1.19 (0.85–1.67)
Relapsed smoker	1.56 (1.01–2.40)	1.62 (1.04–2.50)
**Age**	1.01 (1.00–1.03)	1.01 (1.00–1.03)
**Sex (reference: male)**		
Female	0.98 (0.80–1.19)	1.00 (0.81–1.22)
**Race (reference: white)**		
African American	1.00 (0.82–1.22)	1.06 (0.87–1.30)
Other	1.09 (0.63–1.87)	1.27 (0.73–2.20)
**Insurance type (reference: commercial)**		
Free care	0.87 (0.54–1.40)	0.91 (0.56–1.48)
Medicaid	1.26 (0.54–2.93)	1.34 (0.57–3.16)
Medicare	1.29 (0.97–1.72)	1.25 (0.94–1.67)
Self-pay	0.98 (0.35–2.70)	1.12 (0.41–3.10)
**Type 2 diabetes mellitus outpatient visits (reference: 0–4)**		
≥5	1.33 (1.10–1.63)	1.36 (1.11–1.66)
**Type 2 diabetes mellitus inpatient visits (reference: 0)**		
≥1	1.82 (0.59–5.67)	2.41 (0.77–7.60)
**Charlson Comorbidity Index^a^ (reference: 0)**		
1	1.41 (1.08–1.86)	0.90 (0.62–1.31)
≥2	1.01 (0.79–1.29)	0.79 (0.59–1.05)
**Congestive heart failure (reference: no)**		
Yes	1.65 (1.24–2.21)	1.77 (1.25–2.52)
**Peripheral vascular disease (reference: no)**		
Yes	0.65 (0.31–1.38)	0.66 (0.30–1.42)
**Cerebrovascular disease (reference: no)**		
Yes	1.19 (0.62–2.31)	1.34 (0.66–2.73)
**Chronic pulmonary disease (reference: no)**		
Yes	1.82 (1.34–2.48)	1.92 (1.32–2.80)

a Reference 21.

After analyzing sex and controlling for other variables, we determined that men who were relapsed smokers had a significantly higher risk of diabetes-related ED visits compared with men who were nonsmokers (aHR, 2.05; 95% CI, 1.13–3.71) (Table 3). We also observed that the adjusted risk for women who were continuing smokers was significantly higher than that for women who were nonsmokers (aHR, 1.65; 95% CI, 1.10–2.47). Despite there being no significance for relapsed smokers compared with nonsmokers among the women, results showed an increased risk tendency of ED visits for diabetes when the adjusted model was used (aHR = 1.20, 95% CI, 0.61–2.35).

**Table 3:** Stratification by Sex to Compare the Risk of Emergency Department Use for Type 2 Diabetes Mellitus Across Smoking Status in Cox Models

Smoking status	Women		Men	
	Crude Hazard Ratio (95% Confidence Interval)	Adjusted Hazard Ratio (95% Confidence Interval)	Crude Hazard Ratio (95% Confidence Interval)	Adjusted Hazard Ratio^a^ (95% Confidence Interval)
Former vs nonsmoker	1.35 (0.74–2.48)	1.27 (0.69–2.35)	0.50 (0.18–1.35)	0.47 (0.17–1.29)
Continuing vs nonsmoker	1.68 (1.14–2.47)	1.65 (1.10–2.47)	0.63 (0.33–1.19)	0.67 (0.35–1.29)
Relapsed vs nonsmoker	1.14 (0.59–2.22)	1.20 (0.61–2.35)	2.04 (1.15–3.62)	2.05 (1.13–3.71)

a Adjusted for age, race, insurance type, type 2 diabetes mellitus outpatient visits, diabetes mellitus inpatient visits, Charlson comorbidity index (21), congestive heart failure, peripheral vascular disease, cerebrovascular disease, and chronic pulmonary disease.

## Discussion

This study used the LSU HCSD data to examine the association between smoking and diabetes-related ED visits among patients aged 65 years or older with diabetes. The findings of this study revealed that 11.9% of older patients with diabetes are current smokers (including those who are continuing smokers or relapsed smokers). The current smoking rate in our study is slightly higher than that from the National Health Interview Survey (8.5%) (25). This high smoking rate for older patients with diabetes might be attributable to certain characteristics of patients who seek care in LSU hospitals. LSU HCSD serves large groups of Louisiana’s uninsured and low-income citizens (13). Low-income populations have higher rates of tobacco use (26). Additionally, a previous study using this data set reported that the smoking rate was around 31%, which is higher than the state’s average (13). Our study demonstrates that older patients with diabetes who are relapsed smokers possess a significantly higher risk of diabetes-related ED visits than those who are nonsmokers. These findings are consistent with prior literature showing an association between smoking and increased ED use in populations with other chronic diseases, such as asthma (27). One study pointed out that patients with diabetes who are smokers had a higher average hemoglobin A_1c_ and higher insulin resistance than those who are nonsmokers (28), which supports our findings that older patients with diabetes who are smokers have an increased risk of diabetes-related ED visits. Therefore, we suggest that patients who are relapsed or continuing smokers should stop smoking to reduce the risk of ED use for diabetes.

Our data also suggest that older patients with diabetes who had had 5 or more outpatient visits for diabetes in a calendar year should be more diligent regarding follow-up care. These patients had a significant risk of increased diabetes-related ED visits compared with patients with fewer than 5 outpatient visits. In addition, older patients with diabetes with comorbidities of CHF or CPD should be especially mindful of diabetes management because of our finding of an increased risk of ED use in these populations. In terms of a stratified analysis by sex, the data suggest that a higher risk of diabetes-related ED visits is associated with men with diabetes who are relapsed smokers and women with diabetes who are continuing smokers. As a result, these patients may represent major target populations for smoking cessation interventions, which may significantly lower their risk of diabetes-related ED visits.

Our study demonstrates that smoking plays an important role in increasing the risk of diabetes-related ED visits among older patients with diabetes. These findings also suggest that smoking cessation is favorable for both patients and health care systems. Therefore, we recommend designing more efficient smoking cessation interventions for senior smokers with diabetes to increase their motivation to quit, thereby improving their rate of smoking cessation. These interventions include smoking cessation programs (29), diabetes management counseling and other forms of treatment as a component of diabetes care (12), and cessation advice from physicians (30).

This study has some limitations. First, the electronic health care record used by LSU HCSD is designed to remind physicians to ask their patients’ smoking status every 90 days rather than at each outpatient visit. Therefore, we only included patients with more than 2 time points of recorded smoking status that were more than 90 days apart. Additionally, in this study we defined “nonsmokers” as never smokers and smokers who had already quit smoking for more than 1 year. This might have limited the generalizability for these patients. Second, the data did not include information on number of cigarettes consumed. Therefore, this study cannot identify patients’ nicotine dependence. Third, we could not obtain patients’ educational level or household income, which may have affected ED use. However, we considered insurance type, which might be associated with number of ED visits (5). Moreover, this study does not capture ED use and other health care use that did not occur in the LSU public hospital system. The results in this study may therefore underestimate patients’ actual ED use. Lastly, the patient population that the hospital system predominantly serves could further affect the generalizability of the results.

Although some limitations exist, there are several strengths in this study. First, this study applied a longitudinal design to present stronger evidence of an association between smoking status and diabetes-related ED visits. Second, the large EHR databases used in this study reduce the effect of recall or self-report bias, thereby delivering results that are more valid than those from surveys (20). Third, we focused on the association between smoking status and diabetes-related ED visits and used criteria to identify patients’ smoking status regarding their smoking behavior in the past 30 days and 1 year. These criteria were then used to more precisely reflect patients’ actual smoking behavior and examine the association between smoking status and ED visits for diabetes. Finally, previous literature has only explored factors that are associated with quitting smoking after the diagnosis of diabetes. Our study provides empirical evidence of a higher risk of diabetes-related ED visits among older patients who are relapsed smokers.

Our study shows that older continuing and relapsed smokers with diabetes have a higher risk of diabetes-related ED visits than do younger continuing and relapsed smokers. From a primary care perspective, we recommend that primary care physicians provide more intensive diabetes management strategies for these patients to decrease disease progression. In addition, older smokers with diabetes should be encouraged to quit smoking to improve diabetes management.
